# Investigation of the Occurrence of Nighttime Topside Ionospheric Irregularities in Low-Latitude and Equatorial Regions Using CYGNSS Satellites

**DOI:** 10.3390/s20030708

**Published:** 2020-01-28

**Authors:** Liang Huang, Yi Liu, Qiong Tang, Guanyi Chen, Zhuangkai Wang, Chen Zhou

**Affiliations:** Department of Space Physics, School of Electronic Information, Wuhan University, Wuhan 430072, China; 2015102120013@whu.edu.cn (L.H.); liuyiwhuhan@whu.edu.cn (Y.L.); qiongtang@whu.edu.cn (Q.T.); chenguanyi@whu.edu.cn (G.C.); wangzhuangkai@whu.edu.cn (Z.W.)

**Keywords:** CYGNSS, nighttime topside ionospheric irregularities, wavenumber 4 pattern, DE3

## Abstract

By using multi-satellite observations of the L1 signal-to-noise ratio (SNR) from the Cyclone Global Navigation Satellite System (CYGNSS) taken in 2017, we present the occurrence of nighttime topside ionospheric irregularities in low-latitude and equatorial regions. The most significant finding of this study is the existence of longitudinal structures with a wavenumber 4 pattern in the topside irregularities. This suggests that lower atmospheric waves, especially a daytime diurnal eastward-propagating zonal wave number-3 nonmigrating tide (DE3), might play an important role in the generation of topside plasma bubbles during the low solar minimum. Observations of scintillation events indicate that the maximum occurrence of nighttime topside ionospheric irregularities occurs on the magnetic equator during the equinoxes. The current work, which could be regarded as an important update of the previous investigations, would be readily for the further global analysis of the topside ionospheric irregularities.

## 1. Introduction

Topside ionospheric irregularities observed in low-latitude and equatorial regions, which originate in the unstable bottomside ionosphere, have been observed for many years [1,2,3,4,5]. A deep plasma density depletion that occurs at night is called a plasma bubble. Plasma bubbles are generated in the bottomside of the equatorial *F* region and extend vertically to the topside ionosphere. They are caused by the nonlinear plasma process of the generalized Rayleigh-Taylor (R-T) instability [6]. Statistical studies of the occurrence of topside ionospheric irregularities in low-latitude and equatorial region have been conducted over the last few decades. Oya et al. [7] found that topside ionospheric irregularities mainly occurred after sunset. Su et al. [8] proposed that the occurrence of topside ionospheric irregularities was dependent on geomagnetic and solar activity. The latitudinal, longitudinal, and seasonal characteristics of topside ionospheric irregularities were investigated by Kil and Heelis [9].

Longitude/season-dependent structures exhibiting topside ionospheric irregularities in low-latitude and equatorial region have been intensively studied [10,11,12,13]. Su et al. [8] and Li et al. [4] determined the influence of evening equatorial ionization anomalies (EIA) and pre-reversal ***E*** × ***B*** drifts on the longitude variation of topside ionospheric irregularities. Distinct from the electrodynamic control of topside ionospheric irregularity generation, the effects of atmospheric waves on the distribution of plasma bubbles are still unclear and require further investigation.

Statistical analyses of satellite observations from the Communications/Navigation Outage Forecasting System (C/NOFS) during solar minimum (2008–2009) revealed the existence of a wave-4 structure in the longitudinal plasma density irregularity distribution [14]. Dao et al. [14] suggested that a tidal wind-generated daytime zonal electric field could produce a more favorable condition for the R-T instability, which means that density irregularity generation can be modulated by tidal wind. However, by using the ROCSAT-1 observations taken during solar maximum (2000–2002), Kil et al. [15] recently pointed out that diurnal eastward-propagating zonal wave number-3 nonmigrating tide (DE3) signatures were not obvious in the equatorial plasma bubble distribution. They calculated the linear growth rate of the R-T instability and proposed that the effects of the daytime DE3 on the generation of plasma bubbles were insignificant during the solar maximum period. They proposed that the magnitude of the evening pre-reversal enhancement (PRE) is a much more significant factor in the generation of plasma bubbles. In contrast, by using multisatellite observations, Sidorova and Filippov [16,17] identified obvious equatorial plasma bubbles with four wave structures and proposed that the initial density perturbation, which is a condition necessary for plasma bubble generation, can be modulated by the DE3, resulting in the four-peak longitudinal distribution of the plasma bubbles.

In previous studies, the global distribution of topside ionospheric irregularities was determined using in situ measurements taken with the plasma density probe and global navigation satellite system (GNSS) receiver on low Earth orbit (LEO) satellites (see [4,8,13,18]). In this study, we utilized the GNSS observations from eight Global Navigation Satellite Systems-Reflectometry (GNSS-R) satellites that orbit at low and equatorial latitudes. Due to their condensed temporal and spatial coverage, these observations provide a unique opportunity to investigate the statistical occurrence of topside ionospheric irregularities.

## 2. Instruments and Data

CYGNSS consists of eight LEO satellites in a single plane at an altitude of about 510 km and an inclination of 35°. These satellites were launched in December 2016. The mission, which is operated by the National Aeronautics and Space Administration (NASA), mainly focuses on making frequent observations of the ocean surface wind field in all precipitation conditions, especially in and near the hurricane eyewall [19]. The delay-Doppler mapping instruments (DDMI) installed in each observatory can measure the direct GPS L1 signals and the signals scattered off the ocean surface. These signals can be used to retrieve the ocean surface wind speed [20]. In this study, the direct GPS signal’s SNR data with a time resolution of 1 s from the CYGNSS L1B dataset for March 18, 2017, to March 17, 2018, are analyzed.

The amplitude fluctuations of the global positioning system (GPS) signals are produced by ionospheric irregularities (Spread F, plasma bubble, etc.), which are defined by amplitude scintillation index *S*_4_ [21]. The *S*_4_ index calculation formula is as follows:

(1) $$S_{4}=\sqrt{\frac{\langle {\left(I-\langle \bar{I}\rangle \right)}^{2}\rangle }{{\bar{\langle I\rangle }}^{2}}}$$

where *I* is the square of the GPS signal’s SNR, and we use a voltage SNR to calculate, and the brackets 〈〉 indicate the average over 60 s. The noise has the same statistical properties as the signal so that the detrending and other operations do not change their ratio, then $\bar{\langle I\rangle }$ is the detrended signal using a low-pass filter. In this study, the GPS receiver on each CYGNSS satellite has four receiving channels, which could simultaneously measure the GPS L1 signals from four different GPS satellites. *S*_4_ index is calculated based on different GPS signals’ SNR, respectively. We assume that the scintillation event occurs during an observational event as long as one of *S*_4_ value satisfies *S*_4_ ≥ 0.2. While those with *S*_4_ ≥ 2 are considered as outliers and removed. Moreover, the geolocation of scintillation event is assigned to the position information of CYGNSS satellites. In this study, the orientation of satellite–receiver link is not taken into account. The reason is as follows. The height range of topside ionospheric irregularity occurrence is about 400–850 km based on the recent observational studies (see [4,11,13,22]), and the orbital altitude of CYGNSS satellites is about 510 km. Thus, the scintillation event caused by topside ionospheric irregularity mainly occurs near CYGNSS satellite altitude. We use only signals from satellites at elevation angles (with respect to the satellite’s horizon plane) greater than zero, e.g., not from transmitters in an occultation geometry, or signals reflected off the ocean’s surface. The effect of the orientation of GPS satellite–receiver link on statistical study of topside ionospheric irregularity occurrence can be neglected.

## 3. Results

**Figure 1:**
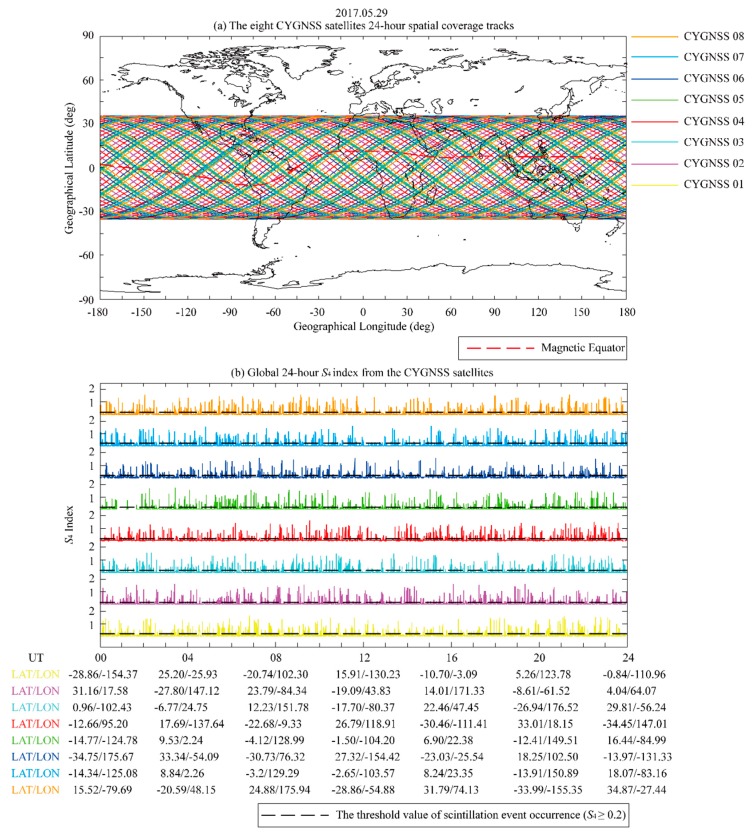
Example of observations taken by the CYGNSS satellites on May 29, 2017. (**a**) The eight CYGNSS satellites 24-h spatial coverage tracks. (**b**) Global 24-h *S*_4_ index from the CYGNSS satellites. The magnetic equator is represented by red dotted line. The black dotted line indicates the threshold value of scintillation event occurrence (*S*_4_ ≥ 0.2).

An example of the observations taken by the CYGNSS satellites on May 29, 2017, is shown in Figure 1. The CYGNSS spatial coverage tracks after 24 h (Figure 1a) suggest that the *S*_4_ index observation events could cover the entire longitudinal sectors within the ±38° latitude coverage region. 

Figure 1b presents the 24-h observations of the global *S*_4_ index. The observation results confirm that topside ionospheric irregularities in low-latitude and equatorial region, which are represented by the scintillation events, can be detected by the CYGNSS satellites.

**Figure 2:**
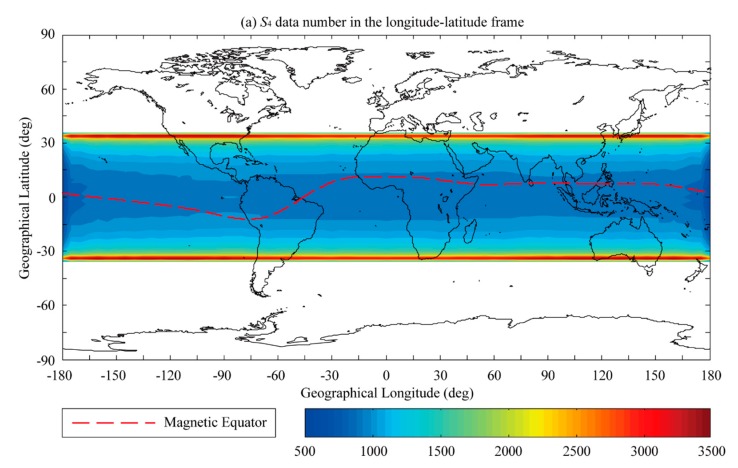

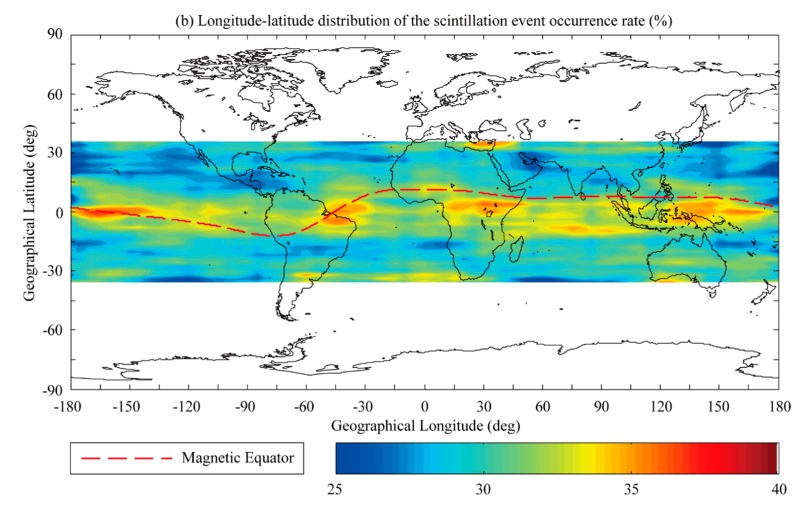
Distribution of the global scintillation events for nighttime (i.e., 18-06 LT) in the 2° × 5° spatial resolution longitude-latitude frame from the CYGNSS satellites in 2017. (**a**) S4 data number in the longitude-latitude frame. (**b**) Longitude-latitude distribution of the scintillation event occurrence rate (%).

Figure 2 shows the distribution of the global scintillation events for nighttime (i.e., 18-06 LT) within a 2° × 5° spatial resolution longitude-latitude frame observed by the CYGNSS satellites in 2017. Only the S4 greater than 0.2 are counted and the S4 which greater than 2 are removed as outliers. As shown in Figure 2a, the distribution of the *S*_4_ data number is generally latitudinally independent within the ±30° region. The temporal resolution is 1 s, and we defined the S4 data number by calculating all the S4 value in the 2 × 2 grid. Due to the satellite orbits, the number of *S*_4_ data is larger within the boundary area, i.e., at latitudes greater than 30°. We have considered the effect of the latitudinal dependence of the calculation on the distribution of the global scintillation events derived from the CYGNSS observations. As can be seen in Figure 2b, the scintillation events exhibit an obvious four-peak longitudinal structure. Figure 2b shows that the occurrence of equatorial scintillation events is more prominent than that in low-latitude region.

**Figure 3:**
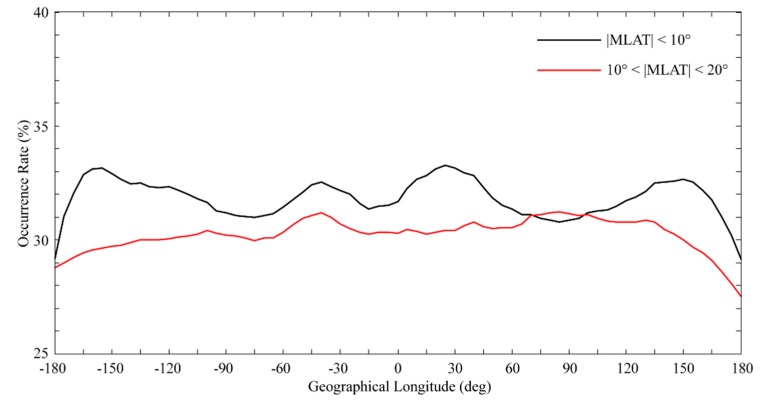
Longitudinal structure of scintillation event occurrences for nighttime (i.e., 18-06 LT) in magnetic low-latitude and equatorial region calculated from the CYGNSS direct GPS SNR data from 2017. The variations in the scintillation event occurrences calculated for magnetic low-latitude ($10^{{}^{\circ}}<\left|\mathrm{MLAT}\right|<20^{{}^{\circ}}$) and equatorial ($\left|\mathrm{MLAT}\right|<10^{{}^{\circ}}$ ) region are represented by the red and black curves, respectively.

Figure 3 shows that the occurrence of the nighttime (i.e., 18-06 LT) scintillation events within magnetic low-latitude ($10^{{}^{\circ}}<\left|\mathrm{MLAT}\right|<20^{{}^{\circ}}$) and equatorial ($\left|\mathrm{MLAT}\right|<10^{{}^{\circ}}$) region is longitudinally dependent. The longitudinal dependence of the wave-4 structure in magnetic equatorial region is also clearly illustrated in Figure 3. At magnetic low-latitudes, one sees activity throughout the South American and East Asian sector and a wave-4 pattern is not evident. The occurrence of scintillation events in magnetic equatorial region is higher than that in magnetic low-latitude region, which is consistent with the results Dao et al. [14] obtained using the ROCSAT-1 satellite data.

**Figure 4:**
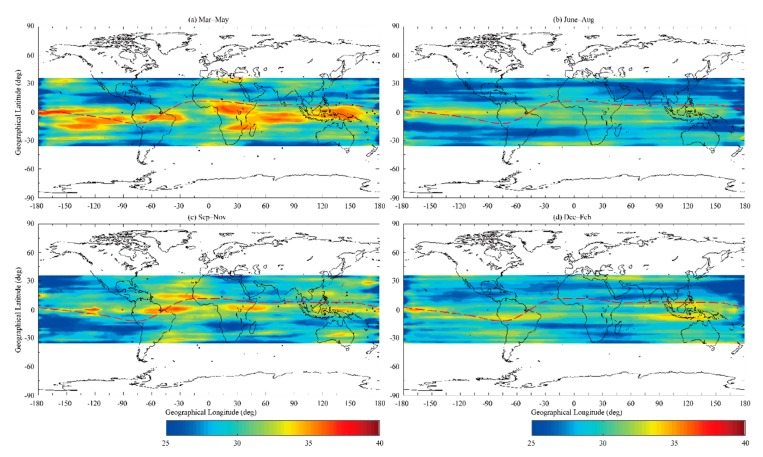
Occurrence rate of the nighttime (i.e., 18-06 LT) scintillation events in different seasons in 2017 binned within a 2° × 5° spatial resolution.

Figure 4 shows the global distribution of the nighttime (i.e., 18-06 LT) scintillation events in different seasons in 2017. It should be noted that wave number-4 longitudinal structures exist during the equinoxes (March–May, and September–November), though not as strong during the solstices (June–August, and December–February). Figure 4 demonstrates that there are more frequent occurrences during the equinoxes than during the solstices. This result is in agreement with those of previous studies (see [4,14]). Moreover, equinoctial asymmetry of scintillation event occurrence was also found in Figure 4. Scintillation events could be more frequently seen in March-May time (northern spring season) compared with those in September-November time (northern autumn season).

## 4. Discussion

In the above analysis, we focused on the statistical occurrence of topside ionospheric irregularities using the *S*_4_ index calculated from the GPS signals from the CYGNSS satellites. Due to the adequate coverage provided by the eight CYGNSS satellites, our statistical results reveal wavenumber 4 longitudinal structures in the global distribution of topside ionospheric irregularities.

For most of the observations, we considered the R-T instability to be the principal mechanism for the generation of topside ionospheric irregularities in low-latitude and equatorial regions. It has been suggested that equatorial ionospsheric irregularities can be excited by initial seeding perturbations in the bottomside of the nighttime equatorial *F* layer where the plasma density gradient is oriented upward [23,24]. The formula for the linear growth rate of an R-T instability presented by Sultan [25] suggests that the evolution of ionospheric irregularities is mainly controlled by the zonal polarized electric fields. However, during the day, any polarized electric fields caused by an instability in *F* region would be shorted in *E* region when the *E* region dynamo dominates, even if the plasma density gradients exist in the bottomside during the day. This electrodynamic process restricts the evolution of the R-T instability, resulting in fewer ionospheric irregularities occurring before sunset [6]. And beside the daytime E-region high conductivity, other important factors include small density gradient in daytime bottomside of the low altitudes F region, and high recombination rate between ionized particles and neutral particles. In addition, Burke et al. [11] and Dao et al. [14] have shown that the greatest number of ionospheric irregularities occurred on the magnetic equator. Plasma bubbles occur in the equatorial region because the Rayleigh-Taylor instability is excited in the equatorial F region. The difference in the vertical plasma drift at different longitudes results in the difference in the occurrence rate of plasma bubbles. Therefore, the number of ionospheric irregularities is greater in magnetic equatorial region. Our results, which are presented in Figure 3, also provide observational evidence of the above theory.

In our statistical results, the nighttime topside ionospheric scintillation events had a maximum occurrence in the equinoxes in low-latitude and equatorial region. As proposed by Tsunoda [26], seasonal maxima of plasma bubble occurrence are consistent with the time of year when the sunset terminator line is aligned with the geomagnetic meridian plane. Moreover, equinoctial asymmetry of ionospheric irregularity occurrence was also shown in Figure 4. Otsuka et al. [27] presented that scintillation event occurrence rate was higher during March-April than that during September-October in 2003–2004 in the equatorial region. By using COSMIC observations at solar minimum, same equinoctial asymmetry of scintillation event occurrence rate in the low-latitude and equatorial region was also shown in Dymond [28]. Maruyama et al. [29] investigated the possible connection between equinoctial asymmetry of ionospheric irregularity occurrence and *F* region meridional wind by using numerical model and three ionosonde data in the equatorial and low-latitude region. They found that the meridional wind speed was larger in September-November time (northern autumn season) compared with that in March-May time (northern spring season) and the growth time of plasma bubble increased with the increase of meridional wind speed. Their results demonstrated the hypothesis proposed by Maruyama and Matuura [30] that the transequatorial wind would suppress the R-T instability. Moreover, the equinoctial asymmetry in ionospheric vertical plasma drift speed and annual variation of plasma density also play a significant role in the generation of equinoctial asymmetry of ionospheric irregularity occurrence in the equatorial and low-latitude region [31,32]. In our observational results, it should be noted that there was a more frequent occurrence in American longitudinal sector than that in Asian longitudinal sector during autumn season, which was agreement with their results shown in Juan et al. [33] by using ground-based GNSS TEC data. Longitudinal dependence of seasonal distribution of ionospheric irregularity occurrence in the low-latitude and equatorial region has been a subject of intense research (see [28,33,34,35]). Dymond [28] presented that seasonal distribution of equatorial ionospheric irregularity occurrence had a clear longitudinal dependence based on COSMIC observations. Brahmanandam et al. [22] proposed that the variations of magnetic declination and geographic latitude of magnetic equator with geographic longitude could explain the longitudinal dependence of seasonal distribution in plasma bubble activity.

Long-term observations of the equatorial spread-*F* from the JULIA radar in Jicamarca suggest that the morphology of the ionospheric irregularities depends on the solar cycle. Plasma bubbles occur earlier in the evening and rise to higher altitudes during increased solar activity [36]. During low solar flux conditions, there are fewer observations of topside ionospheric irregularities in the LEO satellite orbital altitude range, resulting in the lower occurrence rates presented in our study compared with the rate for the solar maximum. The longitudinal structure of the topside ionospheric irregularities during the solar maximum has been studied over the last few decades (see [8,37]). Correlative studies of plasma bubbles and the evening pre-reversal enhancement (PRE) of the zonal electric field at the solar maximum have proposed that the longitudinal/seasonal distribution of topside ionospheric irregularities is dominated by the longitudinal/seasonal variation of the pre-reversal ***E*** × ***B*** drift, which is stronger during higher solar conditions ([4,15]). However, the average solar radio flux index (F10.7) in 2017 was 77 (10^−22^ Wm^−2^Hz^−1^), which indicates a low solar condition. We do not expect the pre-reversal ***E*** × ***B*** drift to have a decisive effect on the global variation of the ionospheric irregularities due to the weaker PRE during low solar conditions. Dao et al. [14] suggested that the DE3 affects the generation of plasma bubbles due to its effect on the daytime ***E*** × ***B*** drift during the solar minimum. It is understood that mapping the zonal polarized electric fields generated by the poleward wind from off-equatorial *E* region to equatorial *F* region plays a critical role in exciting ionospheric irregularities. The daytime diurnal meridional winds simulated by the Global Scale Wave Model (GSWM) presented by Dao et al. [14] indicate that the remarkable four-wave structure in wind field distribution occurred during the equinox, not during the solstice, which provides a reasonable explanation for the observed results shown in Figure 4. In addition, the global distribution of the seeding mechanism, which triggers the R-T instability, is also a significant factor in providing an explanation for the results of our study. And besides this, Kil [38], shows that there is a four-node structure to the vertical plasma drift, too, which will modulate the strength of the Rayleigh-Taylor growth rate as well. Rottger [39,40] proposed that the seeding mechanism may be generated by lower atmosphere gravity waves. However, some investigators (see [40,41,42]) believe that a collisional shear instability plays a key role in generation of the seeding perturbations. In turn, the eastward thermosphere wind plays a crucial role in the development of the collisional shear instability. They claim that the equatorial plasma irregularity production in any longitudinal region of the equatorial ionosphere is primary controlled by eastward neutral wind at sunset. Namely, the larger eastward wind produces the larger growth rate of seed perturbation, the stronger PRE and the larger uplift of the bottom-side F region [42]. According to Sidorova and Filippov (see [16,17]) the tide-induced eastward thermosphere winds can “program” the longitudinal distributions of the future equatorial F region irregularities in the primary moment of their generation, namely, during the seed perturbation development. That is why the four-wave pattern of the topside ionospheric irregularities may be a clear reflection of the picture generated by the initial seeding perturbations modulated by the DE3 tidal waves.

## 5. Conclusions

By utilizing the observations from the CYGNSS satellites in 2017, we statistically investigated the occurrence of topside ionospheric irregularities in low-latitude and equatorial region. We determined the longitudinal structure of the topside ionospheric irregularities. The principal results are summarized as follows:

(1) The statistical occurrence of topside ionospheric irregularities in 2017 is dependent on the season. The greatest number of irregularities occurred on the magnetic equator during the equinoxes.

(2) The topside ionospheric irregularities were seen to exhibit an obvious four-peak longitudinal structure in low-latitude and equatorial region. The effect of the daytime DE3 on the generation of plasma bubbles was significant during the solar minimum.

(3) Compared with the previous work, e.g., Dao et al. [14] and Sidorova et al. [16,17], the joint observation of 8 satellites in our work can be used to detect the irregularity wave-4 structure of ionosphere with a larger space range and a longer time period, which makes the coverage of the observations wider and more reliable. It can be said that we updated the previous method. Our study demonstrates a new and feasible way to investigate topside ionospheric irregularities using the GPS signal’s SNR observations from the LEO satellites.
